# Reasons for Discontinuing Active Participation on the Internet Forum Tinnitus Talk: Mixed Methods Citizen Science Study

**DOI:** 10.2196/21444

**Published:** 2021-04-08

**Authors:** Sanja Budimir, Martin Kuska, Myra Spiliopoulou, Winfried Schlee, Rüdiger Pryss, Gerhard Andersson, Hazel Goedhart, Stephen Harrison, Markku Vesala, Gourish Hegde, Berthold Langguth, Christoph Pieh, Thomas Probst

**Affiliations:** 1 Department for Psychotherapy and Biopsychosocial Health Danube University Krems Krems an der Donau Austria; 2 Knowledge Management & Discovery Lab Faculty of Computer Science Otto-von-Guericke-University Magdeburg Magdeburg Germany; 3 Department of Psychiatry and Psychotherapy Regensburg University Regensburg Germany; 4 Institute of Clinical Epidemiology and Biometry University of Würzburg Würzburg Germany; 5 Department of Behavioural Sciences and Learning Linköping University Linköping Sweden; 6 Department of Clinical Neuroscience Karolinska Institute Stockholm Sweden; 7 Department of Biomedical and Clinical Sciences Linköping University Linköping Sweden; 8 Tinnitus Hub London United Kingdom

**Keywords:** tinnitus, Tinnitus Talk, Internet forum, dropout, reasons for discontinuation

## Abstract

**Background:**

Tinnitus Talk is a nonprofit online self-help forum. Asking inactive users about their reasons for discontinued usage of health-related online platforms such as Tinnitus Talk is important for quality assurance.

**Objective:**

The aim of this study was to explore reasons for discontinued use of Tinnitus Talk, and their associations to the perceptions of Tinnitus Talk and the age of users who ceased logging on to the platform.

**Methods:**

Initially, 13,745 users that did not use Tinnitus Talk within the previous 2 months were contacted and the response rate was 20.47% (n=2814). After dataset filtering, a total of 2172 past members of Tinnitus Talk were included in the analyses. Nine predefined reasons for discontinued usage of Tinnitus Talk were included in the survey as well as one open question. Moreover, there were 14 predefined questions focusing on perception of Tinnitus Talk (usefulness, content, community, and quality of members’ posts). Mixed methods analyses were performed. Frequencies and correlation coefficients were calculated for quantitative data, and grounded theory methodology was utilized for exploration of the qualitative data.

**Results:**

Quantitative analysis revealed reasons for discontinued use of Tinnitus Talk as well as associations of these reasons with perceptions of Tinnitus Talk and age. Among the eight predefined reasons for discontinued use of Tinnitus Talk, the most frequently reported was not finding the information they were looking for (451/2695, 16.7%). Overall, the highest rated perception of Tinnitus Talk was content-related ease of understanding (mean 3.9, SD 0.64). A high number (nearly 40%) of participants provided additional free text explaining why they discontinued use. Qualitative analyses identified a total of 1654 specific reasons, more than 93% of which (n=1544) could be inductively coded. The coding system consisted of 33 thematically labeled codes clustered into 10 categories. The most frequent additional reason for discontinuing use was thinking that there is no cure or help for tinnitus symptoms (375/1544, 24.3%). Significant correlations (*P<*.001) were observed between reasons for discontinued usage and perception of Tinnitus Talk. Several reasons for discontinued usage were associated with the examined dimensions of perception of Tinnitus Talk (usefulness, content, community, as well as quality of members’ posts). Moreover, significant correlations (*P<*.001) between age and reasons for discontinued use were found. Older age was associated with no longer using Tinnitus Talk because of not finding what they were looking for. In addition, older participants had a generally less positive perception of Tinnitus Talk than younger participants (*P<*.001).

**Conclusions:**

This study contributes to understanding the reasons for discontinued usage of online self-help platforms, which are typically only reported according to the dropout rates. Furthermore, specific groups of users who did not benefit from Tinnitus Talk were identified, and several practical implications for improvement of the structure, content, and goals of Tinnitus Talk were suggested.

## Introduction

Tinnitus is defined as the perception of sound without an external source and shows considerable heterogeneity [1]. A promising opportunity for the investigation of this heterogeneity lies in using the internet to gather big data through the support forum Tinnitus Talk, which has been continuously running since March 2011; based on Google Analytics statistics, there were about 2 million unique visitors to Tinnitus Talk from March 2019 to February 2020. The Tinnitus Talk support forum also serves as a hub or signpost for patients with other hearing diseases such as Ménière disease, hyperacusis, or hearing loss, as Tinnitus Talk is encountered when searching the internet for initial information relating to already diagnosed problems or new symptoms.

Tinnitus Talk is an internet platform for accessing, sharing, and discussing information related to tinnitus and other hearing diseases (see also Dandage et al [2]). Tinnitus Talk is free of charge and the full content is available without any restrictions for all internet users. However, registration is required for active participation such as for adding comments and discussion posts, as well as for receiving the Tinnitus Talk newsletter and forum notifications. User posts are supervised by Tinnitus Talk administrators on an ad hoc basis and upon users’ requests in terms of good manners, to prohibit commercial activities, avoid aggressive or abusive posting, and to maintain the logical thematic structure of threads (ie, to supervise the correct localization of discussion posts in appropriate threads).

In May 2018 (the time period of this study), the content of Tinnitus Talk included information sorted into six main sections providing visitors and users with general information about tinnitus and similar hearing issues: Tinnitus (including Research News, Support and Treatments), Pulsatile Tinnitus, Hyperacusis & Ear Pain, Off-Topic, Knowledge Base (including Success Stories and Awareness & Fundraising), and Tinnitus Talk (for general announcements). An additional section, Doctor’s Corner, provides users with access to professional support.

Thus, Tinnitus Talk can best be described as a patient online space enabling computer-mediated communication. Tanis [3] identified two groups of users of health-related online fora. First, there are those who benefit from using the forum. These are frequently users who can cope with their problematic situation and encourage other patients via their use of the forum. The second group includes those whose health situation does not get better by using the forum. These users typically use the fora primarily for discussion. As online resources take on a more and more important role in health care, there is an urgent need to investigate their impact scientifically. As emphasized by De Martino et al [4], the overall quality of online health-related information is poor, and the control and regulation of this information are very difficult. However, unverified and misleading information can influence patients’ opinion, confidence of physicians, as well as their perceived quality of care.

There is an enormous wealth of information from health-related fora that can be scientifically investigated [5-7]; however, this type of research is still at a very early stage. One important approach is investigation of the dynamics of users’ presence and active participation in health-related fora. In this context, it is of crucial importance to understand when and why a member of a patient forum discontinues active participation. Users who discontinue using Tinnitus Talk can be classified as inactive users if they return to the forum after a certain period of absence, or as dropouts if they never return to the platform. Previous research on tinnitus self-help interventions showed that the rate of dropout ranged from 0 to 66% [7]. Some very recent studies explored the use of eHealth tools among tinnitus patients (eg, [8-10]). A first attempt toward understanding the characteristics of users who discontinued using Tinnitus Talk was recently made in a master’s thesis published by Hegde [11]. Outside the context of tinnitus, a qualitative study explored the reasons for dropping out of psychotherapy, and found that dissatisfaction with the quality of psychotherapy was the most important factor for patients [12].

The aim of this study was to focus on the lapsed members of Tinnitus Talk in more detail, and to analyze the reasons for discontinued use. Discovering these reasons is necessary for several purposes. First, this feedback could provide an important source for development of the content and structure of health-related fora. Second, identifying patients’ reasons for discontinuing use of Tinnitus Talk enables recognizing particular groups of users for which further membership in the forum is no longer beneficial, becomes counterproductive, or even harmful. By analyzing users’ views on reasons for discontinuing activity in the forum in this citizen science project, original insight into the world of tinnitus sufferers could be obtained. Furthermore, we were interested in determining the association between reasons for discontinuing the use of Tinnitus Talk and perception of the Tinnitus Talk forum. Intuitively, more negative perceptions of Tinnitus Talk could be associated with a higher probability of discontinuing use of the forum. Moreover, we were interested in potential associations between age and perceptions of Tinnitus Talk and reasons for discontinuing use, since older participants might have more problems with using the internet than younger participants [8,9]. Accordingly, our research questions were as follows: (1) What are the reasons for discontinuing the use of Tinnitus Talk? (2) Are users’ perceptions of the Tinnitus Talk forum related to discontinuing the use of Tinnitus Talk? (3) Is age related to users’ perceptions of Tinnitus Talk or reasons for discontinuing the use of Tinnitus Talk?

## Methods

### Study Design

Tinnitus Hub, a UK-based nonprofit organization that operates Tinnitus Talk, contacted 13,745 users that had not used Tinnitus Talk within the previous 2 months. They were contacted via email, and the study was also announced in newsletters and via forum notifications. The response rate was 20.47% (n=2814). Participants had to complete an English online survey (SurveyMonkey) including questions about perceptions of Tinnitus Talk and reasons for discontinuing use of Tinnitus Talk.

### Participants

Participants of the survey included 2814 individuals who had discontinued use of Tinnitus Talk for at least 2 months. As the answers on the survey questions were not mandatory, the number of answers for each question differed. Participants who did not answer the question “Why did you stop using Tinnitus Talk” were excluded from the analyzed dataset, resulting in a final sample of 2172 participants. There were no significant differences between excluded and included participants in terms of age, gender, status of tinnitus and hyperacusis, and onset of tinnitus or hyperacusis (Table 1).

The sample included mostly male participants with a mean age of 55.70 years. Over 80% of the participants were from English-speaking countries: United States, United Kingdom, Canada, Australia, New Zealand, and Ireland. In general, over 60% of users reported using Tinnitus Talk monthly or even less frequently prior to discontinuing use. Most participants had chronic tinnitus, and the onset of tinnitus symptoms was at least 1-2 years prior for over 91% of the respondents.

**Table 1:** Comparison of characteristics of included and excluded participants.

Characteristic			Included (n=2172)		Excluded (n=642)		Statistic for comparison		*P* value
Age, mean (SD), n		55.70 (14.36), 2157		55.55 (14.89), 497		*t*_2,652_=0.213, *d=*0.01		.83	
**Gender, n (%)**							χ^2^_3_=2.073, critical χ²=7.815, β err prob=0.802, power (1–β err prob)=0.198		.56
	Male	1332 (61.33)		309 (48.13)					
	Female	794 (36.56)		190 (29.60)					
	Other	6 (0.28)		0 (0)					
	Prefer not to say	15 (0.69)		2 (0.31)					
**Diagnosis of tinnitus and/or hyperacusis, n**							χ^2^_1_=0.844, critical χ²=3.841, β err prob=0.849, power (1–β err prob)=0.151		.36
	Yes	2116		491					
	No	49		8					
**Time since onset of tinnitus (n=2659), n**							χ^2^_9_=14.076, critical χ²=16.919, β err prob=0.253, power (1–β err prob)=0.747		.12
	Not applicable	15		4					
	Up to 3 months	21		4					
	4-6 months	47		5					
	6-12 months	110		21					
	1-2 years	275		50					
	2-3 years	320		67					
	3-5 years	400		77					
	5-10 years	338		99					
	10-20 years	307		78					
	More than 20 years	337		84					
**Time since onset of hyperacusis (n=2220), n**							χ^2^_9_=6.624, critical χ²=16.919, β err prob=0.625, power (1–β err prob)=0.375		.68
	Not applicable	1,301		285					
	≤3 months	17		3					
	4-6 months	17		3					
	6-12 months	46		13					
	1-2 years	88		16					
	2-3 years	95		15					
	3-5 years	107		16					
	5-10 years	77		18					
	10-20 years	46		10					

### Instrument

The instrument was a cross-sectional online survey in the English language. The final survey questions resulted from a discussion among members of the research team and with the Tinnitus Talk managers. The research team created questions based on the information provided in a qualitative study on experiences of nonadherence to internet-delivered cognitive behavior therapy [13]. Specifically, one author (TP) developed items based on the results reported by Johansson et al [13] to investigate reasons for discontinued usage of self-help platforms. TP then discussed these items with the coauthors, adjusted them as needed, and sent the final items as suggestions to the Tinnitus Talk manager team. The Tinnitus Talk team then decided which of these items/suggestions they wanted to add to the survey items that they had developed on their own (eg, users’ perception of the Tinnitus Talk forum). The Tinnitus Talk team then asked previous Tinnitus Talk users (no use of the forum for at least 2 months) to complete the final survey instrument. The question of the survey that served as the focus of this study refers to the reasons for discontinued usage. Participants provided reasons for the discontinued usage of Tinnitus Talk by choosing one or more than one answer of a multiple-choice question with eight suggested reasons and an additional open-ended option where participants could list other reasons.

Moreover, we were interested in the relation between discontinued usage and the user’s perception of the Tinnitus Talk forum. Users’ perception of Tinnitus Talk was estimated on a 5-point Likert scale (1=strongly disagree, 2=disagree, 3=neither agree nor disagree, 4=agree, and 5=strongly agree), and included items that measured usefulness, content, community, and quality of forum members’ posts. Three items measured the usefulness (U1, U2, U3), three items measured the content (CN1, CN2, CN3), four items measured the community (CM1, CM2, CM3, CM4), and four items measured the quality of forum members’ posts (Q1, Q2, Q3, Q4).

### Analyses

#### Overview of Mixed Methods Approach

A mixed methods approach was employed. Quantitative analyses were used to examine the reasons for discontinued usage; users’ perception of Tinnitus Talk; and correlations between reasons and perception, reasons and age, and perception and age. The qualitative analysis was employed to explore users’ free-text answers given to the open-ended option on other reasons for discontinued usage of Tinnitus Talk. This type of design prioritizes quantitative analysis followed by a qualitative sequence, providing a complete answer to a research question by including both quantitative and qualitative methods [14].

#### Quantitative Data Analysis

Quantitative data were analyzed in IBM SPSS Statistics for Windows, Version 25.0. Statistical tests (*t* test and χ^2^ test) were performed to evaluate differences between included and excluded participants in terms of age, gender, tinnitus/hyperacusis status, and time since symptom onset. Mean (SD) values were calculated for responses to questions that addressed users’ perception of Tinnitus Talk, and data are presented as frequencies (n, %) for responses to the questions that described reasons for discontinued usage. Multiple-choice answers were dummy-coded for the analyses, with 1 indicating presence and 0 indicating absence of the specific reason. Additionally, the Pearson correlation coefficients (*r*) between age, reasons for discontinued usage, and users’ perception of Tinnitus Talk were calculated with the Bonferroni-corrected adjusted *P* value. All statistical tests were performed two-tailed and significance was judged at *P*<.05.

#### Qualitative Data Analysis

Qualitative data analysis focused on the users’ free-text answers given to the open question on other reasons for discontinuing the use of Tinnitus Talk. This open question generated textual data. The number of characters was not limited. Among the total number of participants (N=2172), almost half (n=1075) utilized this option and provided free-text data for subsequent analysis. The average length of answers was 22 words.

Grounded theory methodology (GTM) [15] was utilized for exploration of the qualitative dataset. GTM allows for generating a theory through the informed and open-minded examination of data. Furthermore, GTM provides a set of systematic coding approaches that supports the formation of a tailor-made category system, with the use of open coding and constant comparison of developed codes [16,17]. GTM is based on the inductive conceptualization of qualitative data. In this study, all of the testimonies (N=1075) written in English and gathered via the SurveyMonkey internet platform were manually coded in a line-by-line manner in the Open Coding and List Coding modes of ATLAS.ti 8.4.4.

The goal of the coding process was to understand and classify the “other reasons” for discontinuing active participation on Tinnitus Talk, which were formulated by the participants as free-text answers. Inductively developed codes were assigned to words, phrases, or entire testimonies to identify the relevant text parts and to label them as the particular answers to the research question (ie, the conceptual framework for the coding procedure was delimited by the research question). The codes were generated to represent the meaning of each answer. The meaning similarity, which was determined by the authors’ subjective judgment, was the key to developing the coding system. The process of establishing codes was gradual in terms of GTM. In some cases, code labels were borrowed from the participants’ own formulations. The partial overlap of free-text answers with predefined reasons was not an issue, because this question allowed for multiple-choice answers. After initial coding of the first 200 testimonies, the coding system was adjusted by two authors (MK and SB) to improve accuracy of the codes and to eliminate subjective evaluation. After this step, previously coded testimonies were revised. Subsequently, the codes were clustered into categories to identify the structure of the dataset.

## Results

### Reasons for Discontinued Usage of Tinnitus Talk

#### Overview of Reasons

Table 2 shows the overall frequencies of reasons selected for discontinued usage, which refer to the percentage of the given answers, as each participant could provide multiple reasons. The most frequently selected predefined reasons for discontinued usage of Tinnitus Talk included not finding what users were looking for (R1), not needing Tinnitus Talk after they found what they were looking for (R2), or no longer needing Tinnitus Talk because their condition improved (R4). A surprisingly high number of discontinued users added free text for other reasons (R9). In total, 1654 particular reasons were identified in their answers, more than 93% of which (n=1544) were inductively coded to investigate the research questions. The remaining (6.65%, n=110) text included other reasons that could not be meaningfully coded and were therefore excluded as miscellaneous. As shown in Table 2, the most frequent other reason was thinking that there is no cure or help for tinnitus symptoms (O6), followed by being busy (O3), avoidance (O2), having positive hope (O4), still using Tinnitus Talk (O8), acceptance/habituation (O1), content issues (O10), improvement (O5), technical issues (O9), and other health issues (O7).

The coding system consisted of 33 thematically labeled codes clustered into 10 categories (Table 3). The description of each category follows, along with some illustrative quotes for each category.

**Table 2:** Users’ reasons for discontinuing the use of Tinnitus Talk.

Question			Participants, n (%)
**Multiple choice question:** **Why did you stop using Tinnitus Talk? (select all that apply) (N=2695)**			
	R1: I could not find what I was looking for on Tinnitus Talk	451 (16.7)	
	R2: I found what I was looking for on Tinnitus Talk and did not need it any more	340 (12.6)	
	R3: I perceived negative effects from using Tinnitus Talk	190 (7.1)	
	R4: I did not need Tinnitus Talk anymore because I improved	297 (11.0)	
	R5: I prefer to share in a face-to-face manner	135 (5.0)	
	R6: Tinnitus Talk was too complicated	93 (3.5)	
	R7: I preferred to use a different forum	45 (1.7)	
	R8: I preferred to use Facebook groups	69 (2.6)	
	R9: Other (please specify)	1075 (39.9)	
**Other (categories based on analyses of free-text answers) (N=1544)**			
	O1: Acceptance/Habituation	140 (9.1)	
	O2: Avoidance	178 (11.5)	
	O3: Busy	212 (13.7)	
	O4: Hope/Positive	160 (10.4)	
	O5: Improvement	92 (6.0)	
	O6: No cure/help	375 (24.3)	
	O7: Other health issues	49 (3.2)	
	O8: Still using Tinnitus Talk	154 (10.0)	
	O9: Technical issues	88 (5.7)	
	O10: Content issues	96 (6.2)	

**Table 3:** Categories and codes for other reasons (R9 in Table 2).

Category	Codes
Acceptance/habituation	Acceptance of tinnitus; Habituation with tinnitus
Avoidance	Avoidance strategy; Reading about tinnitus makes it worse
Busy	Busy, but like to be more active; I’m busy; Lack of time; Some other issues
Hope/positive	Compliments to Tinnitus Talk; Hope expression; New information about tinnitus awaited
Improvement	Improved via something else; Mild tinnitus only
No cure/help	Depression expression; Long time with tinnitus; Negativism of users; No cure, so what; No solution found here; Nothing usable on Tinnitus Talk; Resignation; Tinnitus is back; Tinnitus is too individualized; Tinnitus Talk is not helping
Other health issues	Health issues other than tinnitus
Still using Tinnitus Talk	Did not stop!; I access it occasionally; I’m fine with emails; Not stopped, just paused; Visiting, not logging in
Technical issues	I forgot; Technical issues
Content issues	Vague/outdated info; What to do (advice)

#### No Cure/Help

The most common other reason (375 of 1544 quotes, 24.3%) for why participants discontinued the use of Tinnitus Talk was related to the fact that they considered tinnitus to be an incurable disease, and participants shared a conviction that no help is available for their suffering. The most frequently occurring code in this category was therefore labeled as “Resignation” (72 quotes).

Also the negative attitude of the majority of the community is not helping either, I don’t blame anyone, tinnitus, and other diseases are sad, and it’s completely fine to be sad about it. I am sad about it, everyone’s sad about it, but promoting this sadness won’t help anyone, or at least it won’t help me.

#### Busy

The second highest number of testimonies (212 of 1544 quotes, 13.7%) reflected a personal situation in that participants were fully occupied with other activities such as work duties, important life situations, or some other activities/issues. Under these conditions, spending time on Tinnitus Talk—and sometimes on the tinnitus itself—no longer was a priority, at least for a while.

Testimonies clustered in this category were either very short (eg, “too busy” or “no time”) or provided detailed information about particular activities that led the participants to refocus their attention from suffering from tinnitus to other issues, with the exception of serious health issues, which were sorted in a separate category.

#### Avoidance

The third most common category (178 of 1544 quotes, 11.5%) reflected that a strategy, sometimes reported as based on own experiences of simply not dealing with tinnitus (including avoiding the use of Tinnitus Talk), was the only way to achieve partial relief from tinnitus symptoms. In other words, discontinuing use of Tinnitus Talk was part of an overall avoidance coping strategy:

I miss some of the people from Tinnitus Talk but I decided it was best to avoid it for a while.

#### Hope/Positive

The fourth most common category (160 of 1544 quotes, 10.4%) clustered positive testimonies addressed to Tinnitus Talk (coded as “Compliments to Tinnitus Talk,” 97 quotes), expressions of hope, and expectations of new positive information from the field of research and possible treatment of tinnitus in the future (63 quotes).

I’m now waiting on a scientific breakthrough, when this happens, and a proper treatment is available I will once again share my experiences and learnings. What I have shared certainly has helped others and given them a little hope.

#### Still Using Tinnitus Talk

Importantly, the fifth most frequent category (154 of 1544 quotes, 10.0%) showed that despite the fact that only users who were recognized to have stopped their active participation on Tinnitus Talk were invited to the study, they had actually continued visiting the forum, typically as not logged-in visitors of the website. Codes clustered in this category pointed to the following participants: (i) those who were not aware as to why they were recognized as discontinued users of Tinnitus Talk, (ii) those who reported that they had paused in using Tinnitus Talk but are planning to come back, (iii) those who accessed Tinnitus Talk only occasionally, (iv) those who explicitly reported that they visit Tinnitus Talk but without being logged in, and (v) those who limited their participation to reading emails from Tinnitus Talk.

#### Acceptance/Habituation

This sixth most frequent category (140 of 1544 quotes, 9.1%) represented examples of participants who achieved a state of habituation with tinnitus. In some cases, this achieved habituation was associated with a long duration of tinnitus. The following quote indicated a typical reasoning for deciding to discontinue the use of Tinnitus Talk in this group:

I’ve become habituated to the noise and it no longer bothers me to any extent and staying in a forum just reinforces it as a problem which I’ve now accepted.

Furthermore, in some cases, habituation was characterized by its temporality and fragility, and some participants reported the fluctuation of tinnitus symptoms.

#### Content Issues

The seventh category (96 of 1544 quotes, 6.2%) mirrored the weak points of Tinnitus Talk’s content and operation but also provided a wide scale of valuable recommendations for particular improvements. Approximately half of the testimonies from this category commented on the poor quality of the content of Tinnitus Talk. Information on Tinnitus Talk was considered to be vague or too general, outdated, overwhelming, regurgitated/repetitive, conflicting, untrustworthy, not authoritative, or anecdotal:

One of many shotgun approaches to fixing something on a “maybe this’ll work” basis. I’m not willing to do experiments. It’s depressing enough having the disease.

#### Improvement

Surprisingly, the participants offered many potential improvements of Tinnitus Talk, including a system of evaluation of posts in terms of their helpfulness. The eighth most frequent category (92 of 1544 quotes, 6.0%) comprised mainly individual examples of improvements for tinnitus, and cases of present mild states of tinnitus. A relatively wide scale of solutions for reducing tinnitus symptoms were offered, including the use of hearing aids, attending special tinnitus courses, biofeedback, biomagnetic therapy, neurotin or neuromonics medication, ketogenic diet, and special relaxation sounds on YouTube (eg, rain on a tent, waterfall). It should be kept in mind that these are users’ opinions and are not evidence-based recommendations.

#### Technical Issues

The ninth category (88 of 1544 quotes, 5.7%) consisted of two almost equal parts. First, discontinued use based on technical or formal issues (eg, limited internet access, forgotten password, Tinnitus Talk emails in spam folder, or not being aware they were previous users of Tinnitus Talk). Second, other participants in this group simply articulated the reason of discontinued use as “I forgot.”

#### Other Health Issues

The last category (49 of 1544 quotes, 3.2%) explained that their discontinued use of Tinnitus Talk is based on preoccupation with other health disorders (eg, Ménière disease, cancer) either for themselves or people close to them.

### Users’ Perception of Tinnitus Talk

We analyzed the users’ perceptions of Tinnitus Talk based on items that refer to usefulness (U), content (CN), communication (CM), and quality of the member posts (Q), which were rated on a 5-point Likert scale with 1 representing “strongly disagree” and 5 representing “strongly agree” (Table 4). Overall, *usefulness* was estimated as slightly above average, with participants finding what they needed on Tinnitus Talk (U1). However, they did not find *better quality of information* as compared to other sources (U2), and the fact that Tinnitus Talk provided *information in English* was not considered to be a problem (U3). The highest-rated aspect of the users’ perception of Tinnitus Talk referred to the content, which was rated as *organized, clearly structured* (CN1) and *easy to understand* (CN2). Users also reported not being generally negatively *overwhelmed with the amount of content* (CN3). The users’ perception of the Tinnitus Talk community was estimated on the higher end of average for a *connection to other members* (CM1), *the attitude of most of the members* (CM2), and *feeling welcomed* (CM4). However, *forming connections with positive impact* was not rated very highly (CM3). Estimation of the quality of Tinnitus Talk members’ posts from the perspective of discontinued users included *perception of the helpfulness of the information* provided by forum members (Q1). The *content* was not estimated as negative (Q4). Although there was some *conflicting information* (Q2), the participants also considered that the *provided information is factually correct* (Q3).

**Table 4:** Users’ perceptions of Tinnitus Talk scored on a 5-point Likert scale (1=strongly disagree and 5=strongly agree).

Question			Mean (SD)
**Usefulness (U): How useful was Tinnitus Talk to you as a source of help or information?**			
	U1: I found what I needed on Tinnitus Talk	3.48 (0.86)	
	U2: I found better quality information from other sources	2.74 (0.90)	
	U3: I would have preferred help/information in a language other than English	1.87 (0.95)	
**Content (CN): What did you think of the content of Tinnitus Talk?**			
	CN1: The content was well organized and clearly structured	3.76 (0.69)	
	CN2: The content was easy to understand	3.90 (0.64)	
	CN3: The forum has too much content for me	2.59 (0.88)	
**Community (CM): What did you think of the Tinnitus Talk community?**			
	CM1: I found enough members to relate to or connect with	3.46 (0.82)	
	CM2: I appreciated the attitude of most members	3.72 (0.75)	
	CM3: I made connections that positively impacted me	2.90 (0.89)	
	CM4: I felt welcome	3.65 (0.79)	
**Posts (Q): How did you find the quality of forum members’ posts?**			
	Q1: The advice/information provided by forum members was helpful	3.60 (0.76)	
	Q2: There was conflicting advice/information	3.08 (0.82)	
	Q3: The advice/information provided was factually correct	3.35 (0.66)	
	Q4: I felt the content was negative	2.43 (0.84)	

### Correlations Between Predefined Reasons for Discontinued Usage and Perception of Tinnitus Talk

As participants could choose multiple reasons for discontinuation, we applied dummy coding for this variable; if a reason was selected, it was coded as 1 and otherwise was coded as 0. Significant correlations (Bonferroni-corrected *P*<.001) were found between several predefined reasons (R1-R8 in Table 2) for discontinued usage of Tinnitus Talk and users’ perception of Tinnitus Talk among 135 correlations assessed for the eight predefined reasons (Table 5).

**Table 5:** Correlation between users’ perception of Tinnitus Talk and predefined reasons (N=2124-2157).

Variable		R1^a^	R2^b^	R3^c^	R4^d^	R5^e^	R6^f^	R7^g^	R8^h^	Age
**U1: I found what I needed on Tinnitus Talk**										
	*r*	–0.39	0.23	–0.06	0.22	–0.01	–0.11	–0.02	0.00	–0.18
	*P* value	<.001^i^	<.001	.006	<.001	.71	<.001	.25	.94	<.001
**U2: I found better quality information from other sources**										
	*r*	0.04	–0.05	0.10	–0.01	0.00	0.02	0.09	0.06	–0.07
	*P* value	.07	.04	<.001	.81	.96	.37	<.001	.009	.002
**U3: I would have preferred help/information in a language other than English**										
	*r*	–0.04	0.05	0.06	.04	0.03	–0.01	–0.01	–0.02	–0.17
	*P* value	.06	.02	.009	.06	.19	.69	.74	.43	<.001
**CN1: The content was well organized and clearly structured**										
	*r*	–0.26	0.14	–0.08	0.14	–0.03	–0.20	–0.06	–0.01	–0.11
	*P* value	<.001	<.001	<.001	<.001	.11	<.001	.003	.51	<.011
**CN2: The content was easy to understand**										
	*r*	–0.20	0.12	–0.07	0.12	–0.04	–0.22	–0.05	0.00	–0.11
	*P* value	<.001	<.001	.003	<.001	.09	<.001	.03	.97	<.001
**CN3: The forum has too much content for me**										
	*r*	0.12	–0.05	0.09	–0.07	0.03	0.15	0.05	0.03	0.01
	*P* value	<.001	.02	<.001	.001	.17	<.001	.04	.21	.73
**CM1: I found enough members to relate to or connect with**										
	*r*	–0.30	0.15	–0.05	0.21	–0.05	–0.15	–0.03	0.01	–0.19
	*P* value	<.001	<.001	.03	<.001	.02	<.001	.16	.74	<.001^i^
**CM2: I appreciated the attitude of most members**										
	*r*	–0.17	0.13	–0.19	0.13	–0.03	–0.10	–0.06	-0.01	–0.08
	*P* value	<.001	<.001	<.001	<.001	.17	<.001	.007	.78	<.001
**CM3: I made connections that positively impacted me**										
	*r*	–0.26	0.12	–0.02	0.25	0.01	–0.11	–0.03	0.00	–0.20
	*P* value	<.001	<.001	.31	<.001	.77	<.001	.21	.91	<.001
**CM4: I felt welcome**										
	*r*	–0.25	0.13	–0.03	0.18	–0.03	–0.14	–0.03	0.01	–0.21
	*P* value	<.001	<.001	.25	<.001	.23	<.001	.15	.61	<.001
**Q1: The advice/information provided by forum members was helpful**										
	*r*	–0.30	0.18	–0.11	0.19	–0.03	–0.14	–0.05	–0.01	–0.16
	*P* value	<.001	<.001	<.001	<.011	.23	<.001	.02	.72	<.001
**Q2: There was conflicting advice/information**										
	*r*	0.07	–0.01	0.19	0.01	0.00	0.08	0.00	–0.04	–0.10
	*P* value	.001	.66	<.001	.66	.94	<.001	.95	.08	<.001
**Q3: The advice/information provided was factually correct**										
	*r*	–0.18	0.14	–0.14	0.08	0.00	–0.10	–0.05	0.03	–0.07
	*P* value	<.001	<.001	<.001	<.001	>.99	<.001	.03	.15	.001
**Q4: I felt the content was negative**										
	*r*	0.10	–0.09	0.32	–0.04	–0.03	0.09	0.03	0.00	–0.02
	*P* value	<.001	<.001	<.001	.05	.15	<.001	.13	.87	.42
**Age**										
	*r*	0.15	–0.14	–0.14	–0.28	0.00	0.04	0.05	0.02	—^j^
	*P* value	<.001	<.001	<.001	<.001	.91	.10	.02	.26	—

^a^R1: I could not find what I was looking for on Tinnitus Talk.

^b^R2: I found what I was looking for on Tinnitus Talk and did not need it anymore.

^c^R3: I perceived negative effects from using Tinnitus Talk.

^d^R4: I did not need Tinnitus Talk anymore because I improved.

^e^R5: I prefer to share in a face-to-face manner.

^f^R6: Tinnitus Talk was too complicated.

^g^R7: I preferred to use a different forum.

^h^R8: I preferred to use Facebook groups.

^i^*P*<.001 refers to an adjusted *P<.*000370*=*.05/135 (15×9).

^j^Not applicable.

The reason for discontinued usage of *not finding what they were looking for on Tinnitus Talk* (R1) was negatively correlated with *usefulness* (U1). This indicated that the users’ reason for discontinued usage was associated with the perception of the usefulness of Tinnitus Talk, which included not being able to find what they needed. The same reason was negatively correlated with perception of Tinnitus Talk’s *content,* which was not seen as *well organized and clearly structured* (CN1) or *easy to understand* (CN2), and was positively correlated with the aspect of the content that described the forum *as having too much content* (CN3). One of the explanations of what the users were looking for and could not find on Tinnitus Talk (R1) was revealed by analyzing their perception of communication on the forum (CM). They did not find *enough members to relate or to connect with* (CM1), did not appreciate *the attitude of most members* (CM2), did not make *connections that made a positive impact on them* (CM3), and did not *feel welcome* (CM4). The users also did not find the *quality of the information* they were looking for. This included perception of the advice/information provided by forum members as *not helpful* (Q1), *factually not correct information* (Q3), and *perception of the content as negative* (Q4).

On the other side, there was a group of discontinued users who did find what they were looking for on Tinnitus Talk but subsequently discontinued using the forum (R2). Their reason for discontinued usage was positively correlated with *usefulness* (U1), meaning that they did find what they needed on the forum. They also found the content to be *well organized and clearly structured* (CN1), and *easy to understand* (CN2). The perception of the communication on Tinnitus Talk was generally positive, including *finding enough members to connect with* (CM1), appreciating *the attitude of most members* (CM2), *making connections with a positive impact* (CM3), and *feeling welcome* (CM4). They perceived the quality of the information provided by other members to be *helpful* (Q1), *factually correct* (Q3), and they did not perceive the *content as negative* (Q4).

The correlations between the reason of perceiving negative effects from using Tinnitus Talk (R3) and the perception of the forum clarified their discontinuation of usage. They found *better quality of information from other sources* (U2), did not perceive the forum’s *content as well organized and clearly structured* (CN1), they perceived that it had *too much content for them* (CN3), and they did not appreciate *the attitude of most members* (CM2). Additionally, they did not find advice/information provided by forum members to be helpful (Q1), perceived information as conflicting (Q2) and not factually correct (Q3), and felt that the content was negative (Q4).

Users who discontinued using the Tinnitus Talk forum because their condition had improved (R4) also *found what they needed* (U1). Their perception of the content was positive. They perceived the forum content as *well organized and clearly structured* (CN1), and *easy to understand* (CN2). Their perception of the communication between members was also positive; namely, they found *enough members to connect with* (CM1), appreciated *the attitude of most members* (CM2), made *connections with positive impact* (CM3), and *felt welcome* (CM4). The perceived quality also reflected the satisfaction of these members, as they found the *advice and information provided by other members to be helpful* (Q1) and *factually correct* (Q3).

The reason for discontinued usage of the forum because the users *prefer to share in a face-to-face manner* (R5) or they *prefer to use a Facebook group* (R8) was not significantly correlated with any of the 14 items related to perception of the forum.

The correlation between perceptions of the Tinnitus Talk forum by the users who discontinued because they found it to be *too complicated* (R6) revealed more specific aspects they found to be problematic. First, they could *not find what they needed* (U1). Their perception of the forum’s content was negative because of *not being organized and clearly structured* (CN1), *not easy to understand* (CN2), and *having too much content* (CN3). They did not find *enough members to connect with* (CM1), they did not appreciate *the attitude of most members* (CM2), did not make *connections with a positive impact* (CM3), and they did not *feel welcome* (CM4). They also did not find the *information provided by the forum’s members to be helpful* (Q1), *advice and information were perceived as conflicting* (Q2) and *not factually correct* (Q3), and they felt that *the content was negative* (Q4).

The reason for discontinued usage of the Tinnitus Talk forum because of a *preference to use a different forum* (R7) was positively correlated with finding *better quality of information from other sources* (U2).

### Correlations Between Other Reasons for Discontinued Usage and Perception of Tinnitus Talk

Correlations were also calculated between the 10 qualitatively coded “other” reasons (R9 in Table 2) for discontinued usage and the users’ perception of Tinnitus Talk with a total of 150 correlations assessed (Table 6).

Significant correlations were only found between reasons labeled as “Busy” (O3), “Hope/Positive” (O4), “No cure/help” (O6), “Still using Tinnitus Talk” (O8), and “Content issues” (O10) and users’ perception of Tinnitus Talk. There were no significant correlations between the reasons labeled as “Acceptance/Habituation” (O1), “Avoidance” (O2), “Improvement” (O5), “Other health issues” (O7), and “Technical issues” (O9) with users’ perspective of the Tinnitus Talk forum.

A negative correlation was found between the reason labeled as “Busy” (O3) and the perception of the *content was negative* (Q4), meaning that those who discontinued usage due to being busy did not find the content to be negative. Users who had positive hope (O4) thought that *the content was well organized and clearly structured* (CN1) and that *the advice/information provided by forum members was helpful* (Q1).

The discontinued users who thought that there is no cure or help (O6) could *not find what they were looking for on Tinnitus Talk* (U1). They also did not find the *content to be well organized and clearly structured* (CN1) or *easy to understand* (CN2). Regarding their perception of the other members, they did not find enough *members to relate to or connect with* (CM1), did not appreciate *the attitude of other members* (CM2), did not make *connections that positively impacted them* (CM3), and did not *feel welcome* (CM4). Additionally, the perception of the *quality of the forum* was rated as lower in this group, with not perceiving the advice provided by other members as *helpful* (Q1) or *factually correct* (Q3). In fact, they felt that the content was *negative* (Q4).

The users that stated that they are in fact still using Tinnitus Talk (O8) could *find what they were looking for* (U1). They perceived the content as *well-organized and with a clear structure* (CN1) and there was *not* *too much content* for them (CN3). They found *enough members to relate to* (CM1), appreciated the *attitude of most members* (CM2), made *connections that positively affected them* (CM3), and *felt welcomed* (CM4). This reason was also positively correlated with perceiving the *advice of forum members as helpful* (Q1) and the content was not considered to be *negative* (Q4).

The users who discontinued using Tinnitus Talk because of content issues (O10) could *not find what they were looking for* (U1), and did not find that *the content was well organized and clearly structured* (CN1) or *easy to understand* (CN2). They also did not find *enough members to relate to or connect with* (CM1), did not make *connections that positively impacted them* (CM3), and did not *feel welcome* (CM4). The advice/information provided by forum members was *not helpful* (Q1), there was *conflicting advice/information* (Q2), and *factually not correct information* (Q3).

**Table 6:** Correlations between users’ perception of Tinnitus Talk and other reasons (N=1436-1527).

Variable		O1^a^	O2^b^	O3^c^	O4^d^	O5^e^	O6^f^	O7^g^	O8^h^	O9^i^	O10^j^
**U1: I found what I needed on Tinnitus Talk**											
	*r*	0.07	0.05	0.05	0.08	–0.01	–0.17	0.01	0.16	–0.01	–0.14
	*P* value	.005	.04	.06	.003	.73	<.001^k^	.60	<.001	.79	<.001
**U2: I found better quality information from other sources**											
	*r*	–0.05	–0.02	0.02	–0.04	0.08	–0.01	–0.05	–0.03	0.04	0.04
	*P* value	.06	.46	.53	.10	.002	.72	.08	.25	.10	.08
**U3: I would have preferred help/information in a language other than English**											
	*r*	0.00	0.06	–0.01	–0.03	0.03	0.00	–0.03	–0.03	0.00	–0.02
	*P* value	.88	.02	.58	.30	.22	.87	.19	.29	.99	.35
**CN1: The content was well organized and clearly structured**											
	*r*	0.03	0.03	0.05	0.10	–0.03	–0.11	0.03	0.11	–0.05	–0.15
	*P* value	.26	.20	.05	<.001	.24	<.001	.24	<.001	.07	<.001
**CN2: The content was easy to understand**											
	*r*	0.06	0.04	0.07	0.06	–0.05	–0.10	0.06	0.08	–0.06	–0.13
	*P* value	.03	.09	.01	.01	.05	<.001	.03	.004	.02	<.001
**CN3: The forum has too much content for me**											
	*r*	–0.02	0.03	–0.03	–0.04	–0.01	0.09	–0.05	–0.11	0.00	0.07
	*P* value	.38	.20	.21	.12	.71	<.001	.07	<.001	.86	.01
**CM1: I found enough members to relate to or connect with**											
	*r*	0.05	0.04	0.03	0.07	0.00	–0.12	0.00	0.11	–0.01	_0.13
	*P* value	.08	.15	.32	.006	.93	<.001	.91	<.001	.65	<.001
**CM2: I appreciated the attitude of most members**											
	*r*	–0.01	0.04	0.04	0.07	–0.01	–0.11	0.04	0.11	–0.04	–0.07
	*P* value	.74	.16	.12	.005	.80	<.001	.17	<.001	.18	.006
**CM3: I made connections that positively impacted me**											
	*r*	0.05	0.02	0.01	0.03	0.04	–0.14	0.00	0.10	0.04	–0.13
	*P* value	.08	.54	.65	.19	.09	<.001	.86	<.001	.13	<.001
**CM4: I felt welcome**											
	*r*	0.04	0.03	0.01	0.06	0.03	–0.11	0.02	0.14	–0.05	–0.12
	*P* value	.11	.20	.58	.02	.23	<.001	.47	<.001	.04	<.001
**Q1: The advice/information provided by forum members was helpful**											
	*r*	0.09	0.06	0.08	0.10	–0.01	–0.20	0.02	0.11	–0.02	–0.19
	*P* value	.001	.02	.003	<.001	.68	<.001	.38	<.001	.51	<.001
**Q2: There was conflicting advice/information**											
	*r*	–0.06	0.04	–0.08	–0.02	0.04	0.07	–0.03	–0.05	–0.03	0.13
	*P* value	.03	.13	.002	.56	.16	.007	.26	.06	.21	<.001
**Q3: The advice/information provided was factually correct**											
	*r*	0.03	–0.01	0.05	0.05	–0.02	–0.11	0.02	0.05	0.02	–0.10
	*P* value	.29	.72	.05	.06	.40	<.001	.45	.05	.40	<.001
**Q4: I felt the content was negative**											
	*r*	0.00	0.08	–0.10	–0.01	0.00	0.13	–0.03	–0.11	–0.01	0.05
	*P* value	.97	.001	<.001	.64	.98	<.001	.22	<.001	.83	.06
**Age**											
	*r*	–0.06	–0.15	0.08	–0.06	0.01	0.04	0.06	–0.01	0.05	0.03
	*P* value	.02	<.001	.001	.01	.68	.09	.03	.68	.05	.33

^a^O1: acceptance/habituation.

^b^O2: avoidance.

^c^O3: busy.

^d^O4: hope/positive.

^e^O5: improvement.

^f^O6: no cure/help.

^g^O7: other health issues.

^h^O8: still using Tinnitus Talk.

^i^O9: technical issues.

^j^O10: content issues.

^k^*P*<.001 refers to an adjusted *P<*.000333*=*.05/150 (15×10).

### Correlations between Reasons for Discontinued Usage of Tinnitus Talk and Age

#### Correlations Between Predefined Reasons for Discontinued Usage of Tinnitus Talk and Age

As summarized in Tables 5 and 6, older users discontinued usage of Tinnitus Talk because they *could not find what they were looking for* (R1, R2), whereas younger users were more likely to discontinue usage of Tinnitus Talk because they *perceived negative effects* (R3) or because *they improved and no longer needed Tinnitus Talk* (R4).

#### Correlations Between Other Reasons for Discontinued Usage of Tinnitus Talk and Age

The only significant correlation between age and other reasons (R9 in Table 2; 10 qualitatively coded categories in Table 3) was found between age and *avoidance*, with avoidance being more prevalent among younger users (O2).

#### Correlations Between Users’ Perception of Tinnitus Talk and Age

As shown in Table 5, older users *did not find what they needed on Tinnitus Talk* (U1) and would *not prefer information in other languages* (U3). Older users also did not *perceive the content as well organized and clearly structured* (CN1) or *easy to understand* (CN2). They also did not find *enough members to communicate with* (CM1), did not *appreciate the attitude of most members* (CM2), did not make *connections with positive impact* (CM3), and did not *feel welcomed* (CM4). They did not find *information provided by forum members to be helpful* (Q1) but also did not *perceive information as conflicting* (Q2).

## Discussion

### Reasons for Discontinued Usage of Tinnitus Talk

This study assessed a wide range of reasons for why users discontinued use of the health-related internet forum Tinnitus Talk. Moreover, one of the aims of this study was to provide explanation and understanding of these reasons. Previous similarly oriented studies were mainly focused on the measurement of dropout rates from various health-related web-based platforms with very inconsistent findings [7].

We quantitatively analyzed eight predefined reasons for discontinued usage and qualitatively coded the open option for other reasons. The eight predefined reasons can be categorized into three subgroups. The first subgroup included characteristics of the forum as a reason for discontinued usage, such not finding what one was looking for on Tinnitus Talk (R1), perceiving negative effects from using Tinnitus Talk (R3), and perceiving Tinnitus Talk as too complicated (R6), accounting for 27.2% (listed as a reason 734 times among the total of 2695 reasons listed) of the reasons for discontinued usage. The second subgroup included reasons related to no longer needing the forum, either because they already found what they were looking for on Tinnitus Talk (R2) or because their condition improved (R4), accounting for 23.6% (637/2695) of the reasons listed. The third group of reasons referred to the preference of other sources of information, including sharing in a face-to-face manner (R5), preference of a different forum (R7), or preference of using Facebook groups (R8), accounting for only 9.2% (249/2695) of the total reasons. The remaining reasons fall into the group “other” reasons (39.9%, 1075/2695), which were additionally qualitatively analyzed. Based on these results, the Tinnitus Talk forum could improve with respect to these specific aspects that led to discontinuation.

### Users’ Perception of Tinnitus Talk

Additionally, the users’ perception of the Tinnitus Talk forum revealed that the aspects with the lowest scores were a preference for help/information in a language other than English and that the content was negative. The aspects with the highest scores referred to perceiving the content as easy to understand as well as organized and clearly structured, and appreciation of the attitude of most members. The calculated correlations between the reasons for discontinued usage and the users’ perception of Tinnitus Talk revealed associations that provide clearer insight into the relevant characteristic of the forum and its discontinued usage.

### Correlations Among Reasons, Perception of Tinnitus Talk, and Age

The reasons from the first subgroup, that refer to aspects of Tinnitus Talk such as not finding what they were looking for, perceiving negative effects, and perceiving the forum as too complicated, were significantly correlated with aspects of usefulness such as not finding what they want, content aspects, communication aspects, and some of the aspects of the quality of members’ posts. The perception of negative effects from using Tinnitus Talk was significantly correlated with some aspects of usefulness, content, communication, and quality.

The reasons from the second subgroup, those that refer to no longer needing Tinnitus Talk either because of finding what they needed or because their condition improved, perceived positive aspects of usefulness, content, communication, and quality.

The reasons from the third subgroup, referring to a preference of other sources such as face-to-face sharing or Facebook groups, did not have a significant correlation with almost any aspect of users’ perception of Tinnitus Talk, with the exception of a positive correlation between preferring another forum and finding better-quality information from other sources.

Additionally, older users did not perceive Tinnitus Talk as useful and well organized or easy to understand, and did not find there to be good communication or good quality of other members’ posts. Older users were also more likely to stop using Tinnitus Talk because they could not find what they were looking for, whereas younger users discontinued because of perceiving more negative effects from using Tinnitus Talk or because their condition improved. These results imply that Tinnitus Talk might not be the best suited platform for older users, possibly due to a larger amount of online information as they might not be used to that type of information processing.

Analyses of “other” additional reasons revealed that users who discontinued using Tinnitus Talk because they were too busy did not perceive the content as negative. In addition, users who had positive hope perceived the content to be well organized and clearly structured, and that the advice/information provided by forum members was helpful.

The users who discontinued to use Tinnitus Talk because they think that there is no cure or help did not find what they needed on the forum, and did not find that the content was organized well and clearly structured or easy to understand. They also did not find enough members with whom they could relate, did not appreciate the attitude of most members, did not make connections that positively impacted them, and did not feel welcomed. Moreover, they did not find the advice from other forum members to be helpful or factually correct, and they felt that the content was overall negative.

The users who reported that they were still using the forum found what they needed on Tinnitus Talk, thought that the content is well organized and clearly structured, and that there was not too much content. In general, they found the communication with forum members to be positive through connection with other members, appreciated the attitude of most members, made connections with positive impact, and felt welcomed. They also found that the information provided by forum members was helpful and that the content was not negative.

The users who discontinued using the forum because of content issues did not find what they needed, and did not find that the content was well organized and clearly structured or easy to understand. These members showed dissatisfaction in terms of communication with forum members, as they not find enough members to relate to, did not make connections that positively impacted them, and did not feel welcomed. They also did not find the information provided by forum members to be helpful, and they perceived conflicting and factually incorrect information.

**Figure 1:**
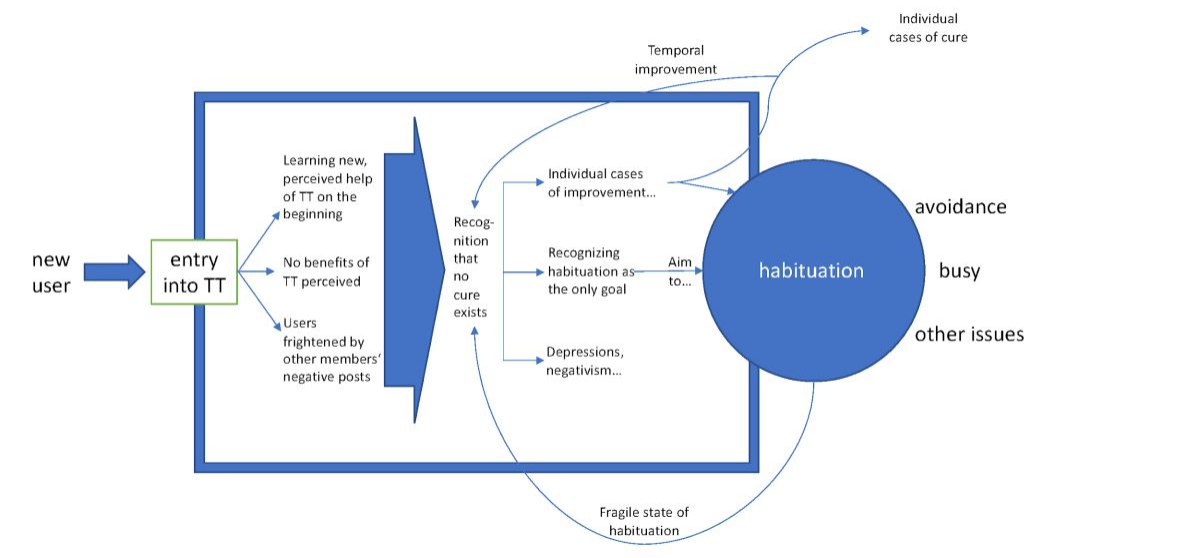
Process model of participation on Tinnitus Talk (TT).

The acceptance of a disease seems to be of crucial importance in the treatment of tinnitus and other chronic diseases [18]. The ability to accept having tinnitus was also understood by some participants as a condition in achieving a state of habituation. To be able to accept tinnitus could be achieved in different ways. Another strategy of how to cope with tinnitus is the avoidance of tinnitus-related themes and thoughts, as reported by participants in this study. This strategy may be helpful in the short-term, but can create problems in the long-term. The avoidance strategy only temporarily helped many of the participants to relieve their symptoms. Although some of them achieved a state of habituation, they reported that this was only for a limited time period (also see Figure 1).

Surprisingly, several valuable recommendations for improvement of the Tinnitus Talk platform were found in testimonies, including ideas to assist users in finding, evaluating, and summarizing the information on Tinnitus Talk, which was recently elaborated by Dandage et al [2]. Some participants of our study reported helplessness related to the strong negativism of several Tinnitus Talk users and their radical posts, which attracted high attention of other users and frightened mainly new users. To maintain and moderate the content is very difficult, especially when the forum is operated on a nonprofit basis.

### Process Model of the Use of Tinnitus Talk

Based on the qualitative analysis, a process model of the use of Tinnitus Talk may be suggested (Figure 1). In testimonies, some participants explained their journey retrospectively from starting as newcomers to Tinnitus Talk with recent onset of tinnitus until becoming a well-informed user who gradually managed to habituate.

The proposed process model tries to reconstruct the experience of Tinnitus Talk users over time. At the beginning, Tinnitus Talk is typically the first or one of the very first resources for tinnitus-related information the user encounters. Gradually, the user’s own tinnitus experience is complemented by accumulated knowledge from Tinnitus Talk. In the quantitative analysis, we found that information saturation (ie, they found what they were looking for but they did not need it anymore) was the second most frequently reported reason for discontinuing the use of Tinnitus Talk. Thus, this process model depicts how Tinnitus Talk works in terms of information saturation among users. Furthermore, it illustrates different pathways of using Tinnitus Talk according to various situations of users. As this study was cross-sectional and we did not assess the time at which a user started using Tinnitus Talk, this model is rather vague. Thus, future longitudinal research is necessary to evaluate this model, which was constructed based on the free-text answers given by only some Tinnitus Talk users.

As we specifically assessed perceptions from a group of lapsed Tinnitus Talk users, there may be many other reasons and motivations to participate in Tinnitus Talk. Health-related fora help a patient cope with their disease, find an important source of social support, feel understood, and allow them to also offer help to other sufferers. The fact that the participants were recruited based on their 2-month absence from the Tinnitus Talk forum is an important limitation of this study. Although this allowed us to more precisely target the group of discontinued users, the perceptions on Tinnitus Talk presented in this study can at best only reflect this group, but cannot be considered as representative for all users of the forum, which comprises approximately 2000 active members. On the other side, complete sampling of lapsed members of Tinnitus Talk was performed and the feedback rate was considerable for an analysis of a group that quit a forum. When interpreting the results, it should also be kept in mind that users of Tinnitus Talk are not representative of other individuals with tinnitus. Previous research has shown differences between Tinnitus Talk users, users of the Track Your Tinnitus smartphone app [19], and patients at an outpatient tinnitus center [20]. According to the criteria applied for the selection of participants, we still detected some users who in fact did not discontinue the use of Tinnitus Talk but are rather taking a break; in other words, they are lurking in more or less a passive way for new information such as a breakthrough in new possibilities of tinnitus treatment.

Another limitation of this study relates to the results of the qualitative analysis, which cannot be generalized, but provide us with a variety of examples that are valuable for understanding individual cases. Moreover, the insight based on participants’ testimonies enables developing new hypotheses for future research. In addition to contributing to understanding users’ reasons for discontinuing the use of a health-related internet forum, several interesting questions for future research have emerged, including (1) How strong is the contagion effect of negativism on various health-related discussion fora in various groups of users? (2) Is there any effect of using Tinnitus Talk on the severity of tinnitus, which can be accessed with the Tinnitus Handicap Inventory [21] (3) What is the accuracy of the health-related advice and recommendations posted by users of Tinnitus Talk when evaluated by medical experts? (4) Does the use of Tinnitus Talk induce some transformation of emotions in various groups of users?

There are also many challenges and calls for qualitative studies in the field, including (1) determining the extent to which users of Tinnitus Talk understand their disease, (2) how they experience their active participation on Tinnitus Talk, or (3) how they perceive the forum to induce or moderate their health-related anxieties or hopes. For future research, it would also be stimulating to link the survey answers of this study to the users’ activities in the forum (ie, behavior, posts, and overall performance on Tinnitus Talk). Unfortunately, this was not possible in this study, which directly targeted only the (presumed) lapsed members. However, we investigated a group of users that are usually out of research scope in the health field. This study revealed this particular group of lapsed users as a very important resource of feedback for preventing dropouts, for improvement of health-related internet fora, and for identification of some weak points and potential risks of health-related fora. The identification of these weaknesses and risks could be utilized as a source of opportunities for improvement.

This study brings forth a variety of practical implications. Reasons that led users to discontinue their active participation on Tinnitus Talk offers valuable feedback for providers of the forum as well as for providers of other health-related fora in the following areas: (1) targeting of communication (eg, newsletters, emails) to specific groups of users, with regard to whether they are newcomers or long-time users; (2) optimization of the structure according to various groups of users (eg, content navigation for newcomers, sections structured for people with different degrees of severity and subtypes of diseases); and (3) evaluation of users’ posts by other forum members as well as by doctors/physicians (eg, in terms of relevance, helpfulness, or potential harm; see also Dandage et al [2]). Some proposals for improvement would require a substantial amount of work and sufficient financial resources on the side of Tinnitus Talk nonprofit providers, namely with respect to continuous content edits, preparing thematic summarizations, quick answering of users’ questions, and in “putting the records straight.” However, this last suggestion seems to be particularly relevant for health-related fora in general, because the quality of discussion posts dramatically fluctuates [22-24]. In addition to the potential dissemination of misleading health-related information, the high occurrence of negativism in users’ posts was considered by some users as a call for a reaction or intervention from the side of Tinnitus Talk providers.

## Abbreviations

CM: communication
CN: content
GTM: grounded theory methodology
Q: quality of member posts
U: usefulness
